# Making sense of evidence in management decisions: the role of research-based knowledge on innovation adoption and implementation in healthcare. study protocol

**DOI:** 10.1186/1748-5908-7-22

**Published:** 2012-03-21

**Authors:** Yiannis Kyratsis, Raheelah Ahmad, Alison Holmes

**Affiliations:** 1 National Centre for Infection Prevention and Management, Faculty of Medicine, Imperial College London, London, UK

**Keywords:** Evidence, Sensemaking, Innovation adoption, Infection prevention, Qualitative, National Health Service (NHS), Hospital, Implementation

## Abstract

**Background:**

We know that patient care can be improved by implementing evidence-based innovations and applying research findings linked to good practice. Successfully implementing innovations in complex organisations, such as the UK's National Health Service (NHS), is often challenging as multiple contextual dynamics mediate the process. Research studies have explored the challenges of introducing innovations into healthcare settings and have contributed to a better understanding of why potentially useful innovations are not always implemented in practice, even if backed by strong evidence. Mediating factors include health policy and health system influences, organisational factors, and individual and professional attitudes, including decision makers' perceptions of innovation evidence. There has been limited research on how different forms of evidence are accessed and utilised by organisational decision makers during innovation adoption. We also know little about how diverse healthcare professionals (clinicians, administrators) make sense of evidence and how this collective sensemaking mediates the uptake of innovations.

**Methods:**

The study will involve nine comparative case study sites of acute care organisations grouped into three regional clusters across England. Each of the purposefully selected sites represents a variety of trust types and organisational contexts. We will use qualitative methods, in-depth interviews, observation of key meetings, and systematic analysis of relevant secondary data to understand the rationale and challenges involved in sourcing and utilising innovation evidence in the empirical setting of infection prevention and control. We will use theories of innovation adoption and sensemaking in organisations to interpret the data. The research will provide lessons for the uptake and continuous use of innovations in the English and international health systems.

**Discussion:**

Unlike most innovation studies, which involve single-level analysis, our study will explore the innovation-adoption process at multiple embedded levels: micro (individual), meso (organisational), and macro (interorganisational). By comparing and contrasting across the nine sites, each with different organisational contexts, local networks, leadership styles, and different innovations considered for adoption, the findings of the study will have wide relevance. The research will produce actionable findings responding to the political and economic need for healthcare organisations to be innovation-ready.

## Background

Health service delivery and organisation as well as clinical practice can be improved through the introduction of novel interventions whose effectiveness is backed by sound evidence. However, the uptake and implementation of innovations in healthcare has often proved challenging and, in some cases, very slow [1,2]. As a result, research findings are not always translated into changes in clinical and managerial practice. This reality also raises the pressing question of how to spread best practice and implement promising innovations within complex settings such as the UK's National Health Service (NHS), a large, professionalised, and highly politicised organisation. The study makes a timely contribution given the recent report *Innovation, Health and Wealth *by the Department of Health on accelerating the pace of adoption and diffusion of innovations in the NHS [3].

Our study is theory based and grounded in the practical experience of healthcare managers to provide answers to persistent challenges. The study draws on organisational theory, largely on sensemaking in organisations, and on innovation theory of change. One of the central questions in this latter body of literature that aligns with the scope of our study is as follows: 'Why do innovations not readily spread, even if backed by strong evidence?' There is a growing body of evidence, including empirical cases in healthcare, that argues that the adoption of new ideas is a process far more dynamic and complex than previously suggested. The classic innovation diffusion model of change, which has influenced UK healthcare policy in the last decades, suggests that the adoption of innovations is a rational and linear process, which is conditioned by the interaction of perceived (by the adopter) innovation attributes with the characteristics of adopters and influences of the social system [4]. This early innovation diffusion work has been criticised, however, for taking a simplistic rational view of change, ignoring the complexities of the change process, and also focusing on individuals rather than organisations. Later work in the same tradition has partially addressed this criticism by explicitly considering adoption *within *organisations [5]. Other innovation and communication studies have departed from the linear model of diffusion [4] to offer conceptual notions that are more dynamic and interactive [6-8]. Building on the latter model, it is suggested that innovation adoption is a process that is highly dependent on the multiple interactions between the innovation, local actors, and complex contextual factors [9-12], including epistemic and social boundaries amongst different professional groups [13].

In addition, the nature or definition of 'evidence' related to innovation is often ambiguous and contested [2,14]. Managers make decisions based on experience, personal expertise, judgment, inference, and advice and do not passively receive new knowledge even if presented as scientifically produced and validated. Research-based knowledge has to be constantly interpreted and reframed along with the local context and clinical or managerial priorities, a process that often involves power struggles among various professional groups [15]. Different professional and managerial groups may interpret evidence differently or may prioritise dissimilar types of evidence partly as a result of their disparate organisational role, education, and training during their socialisation to the profession. We employ a sensemaking perspective to gain insight to this inter- and intraprofessional level and how this plays out in innovation adoption and implementation [16]. This lens pays particular attention to the social construction and coproduction of evidence through the interaction of a range of diverse professional and managerial groups. Our empirical setting of infection prevention and control comprises a multiprofessional workforce operating in a volatile environment, with infection outbreaks coupled with high media and public attention, as well as regulatory and political influences. This environment thus provides opportunities to contribute to this body of literature, which has been useful in explaining organisational response to critical events in the healthcare setting [17,18].

Our work addresses a significant gap in evidence-based healthcare implementation literature. We respond to the call for more sustained interpretive work, which explores the role and motives of actors and the influence of the organisational context and the social construction of evidence [19]. Overall, we aim to address issues that permeate many stages of the research innovation pathway and, more specifically, will investigate processes that relate to the stages of evaluation, adoption, and diffusion. By contributing to the theoretical and empirical discussion, our study will add to the current international and NHS policy and practice body of knowledge. Our work also complements recent and ongoing research commissioned by the National Institute for Health Research Service Delivery & Organisation (NIHR SDO) programme; in particular, it fits well the NIHR SDO 2008 call for proposals that also focused on issues of knowledge utilisation in healthcare management [20]. We complement and add to this work by looking at different types of decisions in different healthcare settings.

### Aims and research questions

The study aims to investigate how healthcare managers draw upon and make sense of different types and sources of evidence when they make decisions about innovations. We include general managers and 'hybrid managers' (clinicians in a managerial role). Special attention is placed on the role of scientifically produced knowledge and its use by these managers during the decision-making process under conditions of innovation uncertainty. The study design incorporates multiple levels of analysis as follows: (a) to explore the influences of wider 'macro' level contextual dynamics on managers' decision making, (b) to explore decision-making processes at the 'meso' organisational level, (c) to analyse at a 'micro' level the processes by which healthcare managers construct meaning of available evidence and how they might use such evidence when deciding on the adoption or rejection of innovations.

### Our key research questions are as follows

• How do managers make sense of evidence?

• What role does evidence play in management decision making when adopting and implementing innovations in healthcare?

• How do wider contextual conditions and intra-organisational capacity influence research use and application by healthcare managers?

## Methods

### Study design

**Figure 1:**
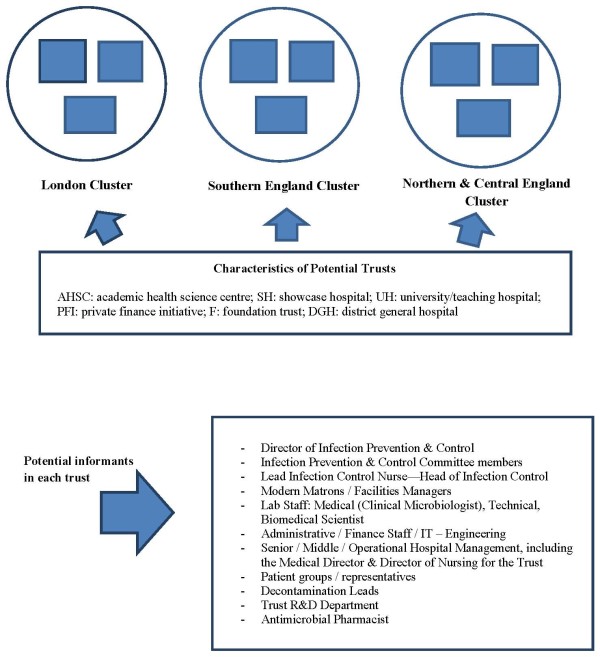
**Field investigation plan: characteristics of sites and sampling of potential informants in each trust**.

The study aims to build theory inductively from multiple in-depth case studies combining inductive search with deductive reason [21,22]. The selection of cases involved theoretical, rather than random, sampling [23]. Nine acute NHS Trusts have been selected across three broad geographic regions in England (Figure 1). Our selected NHS Trusts are equally distributed in three regional clusters: (a) London, (b) Northern and Central England, (c) Southern England. The nine research case studies will be conducted concurrently. By focusing across different localities, we aim to explore the influence of any local network effects if present, for instance, by comparing London-based institutions to non-London-based institutions--bearing in mind the fact that London is a major cosmopolitan city with many healthcare institutions, universities, and research centres, and with a plethora of social and professional events taking place on a regular basis. We anticipate that this potential 'regional effect' may exert influence on the behaviour and perceptions of academics, health professionals, and managers.

In our sample of cases, we include examples of research-engaged healthcare organisations, such as academic health science centres (AHSC) and university/teaching hospitals, and 'ordinary' healthcare service providers, such as district general hospitals. To better delineate the impact of contextual factors in research use and application by healthcare managers on the adoption and implementation of the same innovation, we include multiple 'showcase hospitals' (as selected by the Department of Health to evaluate the in-use value of healthcare-associated, infection-related technologies).

The study will be conducted in two phases, looking in detail at processes in context, first at espoused use of evidence and then evidence use in practice at point of decisions. Phase 1 explores perceptions of senior and operational managers and health professionals in managerial roles across each trust and, specifically, in infection control. Phase 2 explores those organisational members involved in the adoption decisions and implementation of particular technologies in infection prevention and control. We sample for three technologies in each trust fulfilling the following criteria: (a) currently being considered for adoption, (b) successfully adopted and implemented, (c) rejected or discontinued after initial adoption. Using a systematic options appraisal, we will bound the technology by infection prevention control priority area (including hand hygiene, diagnostics, environmental hygiene/cleaning/disinfection, antibiotic prescribing, catheter-related care, training and education, medical devices/equipment hygiene, information technology surveillance systems, patient hygiene) and time frame (of adoption). Technologies considered in the realm of infection prevention and control prior to 2007 will not be included to avoid recall bias and incomplete data due to staff turnover.

### Data collection

The study will last two years and data collection will be longitudinal. Detailed templates will be developed to capture and summarise secondary sources of data and important contextual influences for each of the participating hospitals. This will capture the historical dimension and 'hard' (hierarchy, performance, incentives, control) and 'soft' (espoused values and vision) aspects of organisational culture.

Primary data will comprise semistructured research interviews and research field notes. Some respondents will be interviewed more than once if their organisational role overlaps phase 1 and phase 2 samples. Hence some of the research participants will be involved in the study for two years. Semistructured interview schedules, including short questionnaires with a more structured format, will be developed for the two phases of primary data collection (Additional file 1: Interview Schedule for Phase 1, Additional file 2: Interview Schedule for Phase 2). All data-collection tools will be qualitative in nature and piloted prior to application. The interview accounts will be audio recorded once consent is given by participants.

Informants will include senior, middle, and operational managers; representatives from different professional groups, including medical doctors, infection control specialists, pharmacists, clinical microbiologists, nurses, and allied health professionals; patient representative groups; and nonclinical managers and senior administrators Table 1.

**Table 1:** Sampling of informants, phase 1

**I. Senior members of the infection prevention & control (IPC) team**	Director of IPC (DIPC)
	Deputy DIPC
	Head of IPC
	General Manager IPC
	Lead Infection Control Nurse
	Lead Microbiologist
	Infection Control Physician
	Senior Pharmacist
**II. Middle-range members of the IPC Team**	Decontamination Lead
	Site Managers
	Showcase project managers
	Vascular Access Lead
	< other >
**III. Trust senior executives--clinical**	Director of Nursing
	Medical Director
	Director of Clinical Governance
**IV. Trust senior executives--nonclinical**	Director of Estates & Facilities
	Director of R & D
	Director of Procurement
	Director of Governance
	Head of Outpatients
	Head of Operations

Primary data collection will also include observation of key events. Events sampling will focus on technologies currently being considered for adoption in participating trusts to allow 'real-time' decision-making processes to be explored. Potential events may include infection control team meetings, procurement action group meetings, and trust-based presentation events for new technologies.

Total number of interviews will vary according to the size of the trust and type and span of the innovation. A minimum sample of 6 to 10 key informants per trust is planned, with follow-up interviews and further snowballing to address gaps in the emerging 'story'. Hence, it is estimated that a minimum of 90 to 100 informants will be interviewed overall. The scheduling of interviews over the two-year study will allow opportunities for further snowballing and follow-up interviews with the same respondents where appropriate.

### Data analysis

Soon after the completion of interviews, the content of audio recordings will be transcribed verbatim. Upon completion of transcription, at least three researchers will thoroughly read through the full transcribed text several times to enable understanding of the meaning of data in its entirety [24]. The qualitative data analysis computer software package NVivo 9 (QSR International, Cambridge, MA, USA) will be used to systematically code the collected data and assist analysis. In line with recommendations by qualitative methodologists, we will use multiple coders to enhance interrater reliability of the qualitative study [24,25].

Our qualitative analysis will follow an integrated approach [26]. We will employ an inductive approach to open up new lines of enquiry and then agree on a framework for data analysis based on these findings together with our theoretical framework (delineating factors that influence the adoption process of complex health innovations) and our previous work in 12 NHS Trusts looking at adoption and implementation processes for new technologies [27]. Hence, we will employ both an 'inductive and ground-up' development of codes, as well as a 'deductive organising framework as a start-up list' [26].

Based on the typology suggested by Bradley et al. ([26] p1763), the code types employed in the study are as follows:

(a) Conceptual codes and subcodes: to identify key concept domains and essential dimensions of these domains

(b) Relationship codes: to identify links between other concepts coded with conceptual codes

(c) Participant perspective codes: to identify whether the participant was positive, negative, or indifferent in attitude about a particular experience or part of an experience

(d) Participant characteristic codes: based on professional/occupational group, hierarchical position, functional role

(e) Setting codes: including rural urban setting, hospital site, particular geographic region, type of trust, Strategic Health Authority

The development of the code structure will be finalised when theoretical saturation is achieved in each of the empirical cases [28,29].

Analysis within cases will be followed by the cross-case analysis across emergent themes but also against the more formal organisational 'type' used in our purposeful sampling of sites. Individual case study reports with common formats will be produced as an additional research output for each of the nine trusts. Summary tables will be used to simultaneously compare several categories and dimensions of the content and context of change implied by the adoption and implementation of the innovations across the nine trusts. Pairs of cases as well as groups will be compared by listing similarities and differences in processes, experiences, and outcomes [21].

## Discussion

Our approach allows the application of interpretive methods of inquiry without abandoning the commitment to arrive at a plausible account of commonly shared 'objective' reality. Whilst we uphold that the world and reality do exist independently of the observer, it is not possible for the researcher to escape the social world in order to study the empirical reality [30]. Any given reality can be represented from a range of different perspectives, each perspective being potentially true. This stance presents a possibility of multiple, valid descriptions of the same social phenomenon. We emphasise a more interpretive stance to explore why different understandings and meanings emerge for one observation, and how this explains different views of 'scientific' evidence and when and how different sources of evidence come into play. In addition, we investigate how negotiated orders within teams and organisations validate perceptions and hence lead to sometimes 'hierarchical' views of different sources of evidence.

The potential for learning and generation of new knowledge from this in-depth qualitative study is substantial due to the policy-relevant research aims and fit with methodological approach. We employ a study design of comparative case studies and involve multiple methods, namely, in-depth semistructured interviews, observation of key meetings, short survey questionnaires incorporated in the interviews' topic guide, reflective field notes, and systematic collection and analysis of relevant secondary data from within and outside of the organisations studied. The study is grounded in organisation and management theory, thus enhancing the generalisibility of the findings. We incorporate multilevel analysis exploring influences at micro (individual), meso (organisation/organisational unit), and macro (wider context beyond the organisation) levels and interlevel interactions. Bounding our study in the empirical setting of infection prevention and control facilitates this multilevel analysis. In addition, by employing complementary theoretical approaches, we will triangulate our findings. We will be able to explain how sensemaking occurs when multiple stakeholder groups are involved and the ways in which accounts they generate are--or are not--reconciled [31]. We will situate this within the meso and macro contexts to allow generalisability of our findings.

By combining retrospective and ongoing data, we will explore the social and organisational influences on how evidence is constructed and becomes meaningful to diverse organisational actors. This will allow us to develop a better understanding than short-term and 'snap-shot' methods. As well as the above measures of methodological rigour, we will use measures of acceptability and relevance of the research as defined by key stakeholders, namely, policy makers, academics, clinicians, health managers, and patients. Active involvement of academic experts, health professionals, people with exposure in policy development within the Department of Health, and members of the public through steering-group membership will inform our research. Specifically, our steering-committee members will inform the study design, be involved in the management and oversight of the study, provide feedback on early findings, assist in dissemination of findings to diverse localities and forums, and (through public involvement) advise on 'plain English' summaries of our research outputs.

The current climate of policy and financial uncertainty does pose a challenge in getting access to trusts and informants. We have planned recruitment acknowledging this, and contingency plans have been in place (i.e., we determined an additional pool of potential trusts to replace all types of hospitals initially targeted). We are also mindful of potential challenges in securing interviews over the winter months due to seasonal pressures on hospital staff and the further potential adverse impact of infection outbreaks. We are also aware of the challenges that may arise in coordinating and completing ethical review by NHS sites (completing global and local governance checks across all nine trusts and obtaining letters of access) in time to start data collection as planned. Finally, due to the inductive nature of the study, the number of interviews may vary depending on analytic saturation as well as the nature of technologies sampled. On limitations, a qualitative process study may span a longer duration (greater than 18 months in the field); however, such a project may have posed a higher risk with diminishing returns on research investment given the empirical setting as described above. We do have a longer retrospective dimension in four of the trusts as they comprised a sample from our previous study, which looked at the adoption and implementation processes of novel technologies [27].

## Competing interests

The authors declare that they have no competing interests.

## Authors' contributions

YK and RA conceived the idea for the study and led the intellectual development, funding application, and realisation. AH is the Chief Investigator and YK the Co-Principal Investigator. YK, RA, and AH contributed to the drafting and development of the study. All authors reviewed and agreed on the final manuscript.

## Supplementary Material

Additional file 1 **Phase 1 Interview Thematic Schedule and Questions - Managers**. Click here for file

Additional file 2 **Phase 2 Interview Thematic Schedule and Questions - Adopters and Implementers**. Click here for file
