# Supplementary material for: Making sense of evidence in management decisions: the role of research-based knowledge on innovation adoption and implementation in healthcare. study protocol
Source: Implement Sci. 2012 Mar 21;7:22. doi: 10.1186/1748-5908-7-22 (PMC3325880; doi:10.1186/1748-5908-7-22)
Supplement: Additional file 1 — Phase 1 Interview Thematic Schedule and Questions - Managers. [file 1748-5908-7-22-S1.DOCX]

| **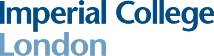** | 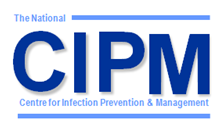 |
| --- | --- |

**Making sense of evidence in management decisions**

**Interview thematic schedule and questions - Managers**

The interview will explore your role and your views on

(a) Innovation adoption in your unit and trust

(b) The use of ‘evidence’ to make such decisions

(c) The different types and sources of ‘evidence’ available to professionals

*All questions apply to your trust about decision making regards innovations, and we are interested in your perceptions. If you do not feel you are able to comment please say so.*

*Do you have any questions before we start?*

1. **Project administration**

| Trust: | Researcher: | Date: |
| --- | --- | --- |
| Post: | Email: | Telephone: |
| Consent form attached | Participant Info provided |  |

1. **Respondent profile**

| Employed in trust since: | Previous appointment / organisation: |
| --- | --- |
| Education & training /  professional background: | Management qualifications: |
| Previous roles within trust: | Membership of professional bodies: |

1. **Respondent role in innovation adoption process**

| Can you please summarise your current role and responsibilities in relation to innovation adoption in the trust. By adoption we mean the decision to introduce an innovation.  Current role in innovation (technology) adoption: | | |
| --- | --- | --- |
|  | **Formal organisational role** | **Informal/**  **other role** |
| Proposing innovations/solutions |  |  |
| Managing budgets in relation to funding innovations |  |  |
| Scanning the environment to identify potential technologies for consideration |  |  |
| Critiquing range of technologies/innovations and associated evidence |  |  |
| Involved in sustaining the innovation/technology & contributing to its continuous use |  |  |
| Involved in decisions to adopt innovations |  |  |
| Seeking or critiquing the principles and mechanisms on which the innovation is based |  |  |
| Involved in critiquing how the innovation works in practice |  |  |
| Involved in persuading others to adopt or reject a particular innovation |  |  |
| Involved in planning and executing |  |  |
| implementation |  |  |
| Do the above roles form part of your formal organisational role? Yes/ No  Can you say which ones ***(go through above list)***  If No – how has your involvement come about? | | |
| Are you a member of any trust wide innovation forums, if there are any? | | |
| Are you a member of any regional/national forums in which innovations & their associated evidence are reviewed or evaluated; either in your organisational role or another capacity? Yes/No  Have you been previously involved in such?  Please tell me about this. | | |

1. **Use of evidence**

- **What do you consider as evidence? What counts as evidence for you?**

1. **Source of evidence**

| If you need to make a decision about selecting an innovation what are your main sources of evidence in relation to innovations?  ***(go through checklist below)***   \| Peer review journals  ***(ask them to say which ones)*** \| Academic  Practitioner  Clinical  Managerial \| \| --- \| --- \| \| Text books \| Management  Clinical \| \| Professional networks \| Mass media (newspaper etc.) \| \| Internal updates (email) \| Own work experience \| \| Other healthcare organisations \| Peers/colleagues \| \| Industry / suppliers \| Management consultants \| \| Department of Health / other arms’ length health agencies eg. NHS Evidence \| Academic institutions/research bodies \| \| Electronic databases *(say which ones)* \| Own knowledge (from training or education) \| |
| --- | --- | --- | --- | --- | --- | --- | --- | --- | --- | --- | --- | --- | --- | --- | --- | --- |

1. **Types of evidence**

| When considering new ideas, practices or products are there different types of evidence you rely upon to inform your decision to adopt or reject them?  When considering different types of evidence is there a hierarchy of evidence? |
| --- |
| - Do different colleagues view evidence differently?   - Why might this be the case? - Do you think evidence is used to support best practice or is used selectively to support agendas/decisions in accordance with organisational or personal priorities? |

1. **Access to evidence**

| - Which format of evidence do you find most useful? - Are there times when you find the content of the presented evidence difficult to understand? - Are there times when you find it difficult to relate evidence to practice? - Do you think that different members of the organisation have or need access to different sources of evidence? What are the consequences of that? |
| --- |

1. **Motivation to use evidence**

| What is your key motivation for sourcing evidence?  ***Prompts***  For best decisions  Best patient outcomes  To justify decisions to others  Financial constraints |
| --- |
| Do you think use of evidence in management decisions can directly improve the performance of the trust? Examples? |
| Have you ever changed your view based on evidence? |

1. **Evidence and Infection Prevention and Control**

| How do you judge and monitor your trust’s IPC performance in relation to other (similar) trusts? |
| --- |
| What sources of pressure are there for your trust to improve its IPC performance? |
| To what extent does this incentivize or inhibit the use of evidence within your Trust?  What do you think might help to promote the use of evidence in IPC? |
| Is IPC among the top priorities for your organisation? If so, how is that demonstrated in practice? |

1. **Enablers to innovation / technology adoption**

| If you now focus on a particular ‘successful’ innovation adoption in the trust:  Which factors acted as enablers to innovation / technology adoption?  ***Prompts:*** Organisational form? How does it impact?  User involvement? Which users? Patients’ feedback?  Leadership support  Communication  Staff capacity?  Management commitment to innovation / technology adoption? How is this demonstrated? |
| --- |
| Did use of evidence feature in the decision process?  Did you have confidence in the evidence used in the decision process? Why?  Did it act as a facilitator or a barrier to innovation / technology adoption?  How? |
| What type of evidence was considered?  What was the source of the evidence? |

1. **Barriers to innovation / technology adoption**

| If you now focus on a particular innovation rejection or discontinuance in the trust.   - What factors acted as barriers to innovation / technology adoption?   ***Prompts:*** Organisational form? How does it impact?  User involvement? Which users? |
| --- |
| Did use or lack of use of evidence in decisions act as a barrier to the process of innovation adoption?  How? |
| What type of evidence was considered?  What was the source of evidence? |

1. **Knowledge sharing and communication**

| What are there the challenges in communicating with people within your unit and in the wider trust?  What seems to work and why?  What would help to improve communication (information & knowledge sharing)?  Do you have a strategy to disseminate information regarding innovations within the Trust?  Does the communication format, content or style change according to the people you are communicating with?  How do you communicate evidence to trust members?  Does the organisational structure affect information sharing throughout the organisation?  What method of communication do you prefer; how would you most like to receive information regarding evidence? |
| --- |

1. **Relevance of and generating evidence**

| What prompts conducting research in a particular area? (need to improve service, people’s agendas)  What kind of research would you like to see? (What research is missing?)  ***Prompts:*** *have there been occasions where you have been involved in something that has led to research,*  *or perhaps should have led to research and did not.* |
| --- |

1. **Involvement in research**

| Have you carried out or been involved in any research in the past? |
| --- |
| Are you interested in carrying out research? |
| Are there challenges to you doing research? |

1. **Knowledge and use of central IPC related evidence sources**

| *Are you* ***aware*** *of the following?* | *Have you ever* ***used*** *any of the following as source of evidence?* |
| --- | --- |
| Rapid Review Panel | Rapid Review Panel |
| Smart Ideas | Smart Ideas |
| Showcase Hospitals | Showcase Hospitals |
| Design Bugs Out | Design Bugs Out |
| Smart Solutions | Smart Solutions |
| Product Surgeries | Product Surgeries |
| Cochrane Library | Cochrane Library |
| National Institute for Health & Clinical Excellence (NICE) | National Institute for Health & Clinical Excellence (NICE) |
| NHS National Patient Safety Agency (NPSA) | NHS National Patient Safety Agency (NPSA) |
| National Service Frameworks | National Service Frameworks |
| NHS Evidence | NHS Evidence |
| Centre for Evidence Based Purchasing | Centre for Evidence Based Purchasing |
| NIHR / Service Delivery & Organisation Programme | NIHR / Service Delivery & Organisation Programme |
| Knowledge Transfer Networks | Knowledge Transfer Networks |
| The National Innovation Centre (NIC) | The National Innovation Centre (NIC) |
| The NHS Institute for Innovation and  Improvement | The NHS Institute for Innovation and  Improvement |
| The NHS National Technology Adoption Centre | The NHS National Technology Adoption Centre |
| DH Purchase and Supplies Agency (PASA) | DH Purchase and Supplies Agency (PASA) |
| Clean safe care website | Clean safe care website |
