# Supplementary material for: Making sense of evidence in management decisions: the role of research-based knowledge on innovation adoption and implementation in healthcare. study protocol
Source: Implement Sci. 2012 Mar 21;7:22. doi: 10.1186/1748-5908-7-22 (PMC3325880; doi:10.1186/1748-5908-7-22)
Supplement: Additional file 2 — Phase 2 Interview Thematic Schedule and Questions - Adopters and Implementers. [file 1748-5908-7-22-S2.DOCX]

| **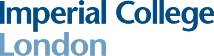** | 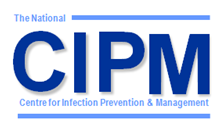 |
| --- | --- |

**Making sense of evidence in management decisions**

**Interview thematic schedule and questions – adopters and implementers**

The interview will explore your role and your views on the process of innovation adoption and implementation regarding << insert particular innovations: successful adoption – rejection/discontinuance – ongoing>>;

*All questions apply to your trust, and we are interested in your perceptions on the following. If you do not feel you are able to comment please say so.*

*Do you have any questions before we start?*

1. **Project administration**

| Trust: | Researcher: | Date: |
| --- | --- | --- |
| Post: | Email: | Telephone: |
| Consent form attached | Participant Info provided |  |

1. **Respondent role**

| Employed in trust since: | Previous appointment /organisation: |
| --- | --- |
| Education & training /  professional background: | Management qualifications: |
| Previous roles within trust: | |

***{the following questions will need to be repeated for each of the following: an innovation technology adopted and implemented successfully; an innovation rejection or discontinuance; an innovation currently under consideration all within the field of environmental hygiene}***

1. **Description and Attributes of the innovation**

| - Could you please briefly describe me the innovation (if information is not available from secondary sources)? - Which of the following best describes the nature of the innovation: - **1. Equipment / Device**  **2. Product**  **3. Protocol**  **4. Process**  **5. Programme**  **6. Guideline** - Is the innovation/technology radically new (new to the NHS) or has it been well established and already validated in other NHS settings? - How does it compare to the pre-existing technologies or pre-established practice that the innovation is intended to replace/support? – is there a clear relative advantage? - Would you describe it ‘high tech’ or ‘low tech’? - What is the perceived anticipated benefit of the innovation/technology to:   - Patients   - Staff (also to different professional; groups)   - Organisation (The Trust)   - The NHS - How complex is the innovation/technology? (to understand its functionality, ease of use, explain to users and other interested actors) - Is the new technology compatible with pre-existing systems / structures / processes – working practices / values and culture in the organisation? |
| --- |

1. **Idea for the innovation**

| - How did the idea for the innovation come about?   **Prompts**  What was the source of information?  Where there presentations (by whom), workshops, visits to other institutions, participation in networks or memberships in professional groups, suppliers of technologies?  What information about the innovation/technology was available through these sources? |
| --- |

1. **Stakeholder involvement**

| - Who was involved in the innovation selection decision?   **Prompts**:  Have you had any involvement in the process? (If yes probe)  Who else was involved? And how?  Members of IPC only – please list  Members outside of IPC – please list  Senior management   - (If group involved), how was consensus achieved? - Has anyone challenged the selection decision? Why? - Are you aware of a champion in promoting the selection and adoption of a particular innovation/technology? - Has the process included seeking views from patients or patient groups? |
| --- |

1. **Decision-making process**

| **What form did the organisational decision making process take?**   - Was the process formal with meetings and minutes? Did the trust form a project team. Was approval required from senior management (i.e. Trust’s board decision, decision made by Heads of Departments, issue discussed in Steering Groups / Committees?) - What was the level of involvement by senior management:   **Prompts:** keeping them informed / approval or disapproval of specific propositions / level of support provided   - Who really decided? (Are you able to say who made the final decision)? - How long did the innovation/technology selection process take?   **Decision-making: selecting environmental hygiene as IPC priority area**   - Was the technology considered first or the IPC area of environmental hygiene? - How did you learn about the range of innovations / technologies available? - What factors were considered for selecting the innovation? - Was sustainability considered in the decision making process? - Were financial pressures a consideration in the decision? - Did staff capacity (numbers and levels of competency) have an influence on decision making? - Did use of evidence feature in the decision making process? - Did the use of evidence act as a facilitator or a barrier to innovation / technology adoption? - How? - What type of evidence was considered? - What was the source of the evidence? - Which factors facilitated or hindered the selection of the innovation?   **Prompts:**   - Individual: personal motivation, champions, sceptics - Intra-organisational: structure, available capacity / skills / resources / alignment with Trust – Department strategies / Trust’s tradition or character - Contextual: Alignment with DH strategies and policy frameworks / Regulative frameworks / pressing societal or health priorities, performance targets |
| --- |

1. **Implementation process**

| - Who was involved in implementation planning? - Which professional and functional/managerial groups were involved in implementation (execution)? - Who were the champions of the innovation / technology? - Have patients been involved / informed in this process? – How? - Have there been any resistors to the innovation implementation? - How easily was it implemented? What have been the facilitating factors and barriers in the adoption and implementation process? - What were the implementation issues for clinicians, NHS managers and others? - What did your trust see as being the measures of success for the sustainable implementation of the innovation? - Was the innovation considered successful by these measures? |
| --- |

***Thank you for your participation in this important research, is there anybody else who you think could provide further insight to the issues discussed today?***
